# Advanced Feature Extraction for Cervical Cancer Image Classification: Integrating Neural Feature Extraction and AutoInt Models

**DOI:** 10.3390/s25092826

**Published:** 2025-04-30

**Authors:** Muhammad Amjad Raza, Hafeez Ur Rehman Siddiqui, Adil Ali Saleem, Kainat Zafar, Afia Zafar, Sandra Dudley, Muhammad Iqbal

**Affiliations:** 1 Institute of Computer Science, Khwaja Fareed University of Engineering and Information Technology, Abu Dhabi Road, Rahim Yar Khan 64200, Pakistan; ch.amjadraza@gmail.com (M.A.R.); hafeez@kfueit.edu.pk (H.U.R.S.); adilalisaleem@gmail.com (A.A.S.); 2 Faculty of Computing, Riphah International University, 2 KM McDonald’s Lahore Multan Bypass Road, Sahiwal 57000, Pakistan; 3 Department of Computer Science, National University of Technology, Islamabad 44000, Pakistan; kainatz@nutech.edu.pk (K.Z.); afiazafar@nutech.edu.pk (A.Z.); 4 Bioengineering Research Centre, School of Engineering, London South Bank University, 103 Borough Road, London SE1 0AA, UK; dudleyms@lsbu.ac.uk; 5 School of Interdisciplinary Engineering and Sciences, National University of Sciences and Technology, Islamabad 44000, Pakistan

**Keywords:** cervical cancer, Neural Feature Extractor, VGG16, AutoInt

## Abstract

Cervical cancer remains a significant global public health challenge, affecting over half a million women annually, with a mortality rate of approximately 60%, especially in resource-limited regions. This study presents an advanced methodology for cervical cancer diagnosis through deep learning techniques. Utilizing a publicly available cervical cancer image dataset, the research introduces a novel classification framework that integrates a Neural Feature Extractor (NFE) based on a pre-trained VGG16 architecture and an AutoInt model for automatic feature interaction learning. The extracted features are processed through machine learning classifiers such as KNN, LGBM, Extra Trees, and others for classification tasks. Among these classifiers, KNN achieved the highest accuracy of 99.96%, followed closely by LGBM at 99.92%. This study also assesses the computational complexity of various models, demonstrating that simpler models like LDA exhibit faster prediction times, while more complex models, such as KNN and LGBM, provide higher accuracy. These findings highlight the potential of deep learning frameworks in improving cervical cancer classification accuracy, especially in resource-limited environments.

## 1. Introduction

Cervical cancer presents a substantial public health dilemma, with an annual diagnosis rate of over 500,000 women and resulting in more than 300,000 fatalities globally [1,2]. Ranked as the second most prevalent cancer among women globally, cervical cancer carries a substantial mortality rate of 60%. Alarmingly, approximately 85% of the annual fatalities occur in developing countries, where medical resources, including professionals and technology, are notably limited [2,3,4]. The majority of these deaths could be averted with widespread access to routine screening tests, facilitating the timely treatment of precancerous lesions [5,6]. Given the elusive nature of early-stage cervical cancer symptoms and its prolonged latent period, regular checkups play a crucial role in early detection and intervention [7,8]. Long-term infection with the sexually transmitted Human Papillomavirus (HPV) is the primary cause of cervical cancer. While the majority of sexually active individuals will acquire HPV, only a small percentage of women will ultimately develop cervical cancer, as influenced by additional factors [9].

The traditional method for detecting cervical cancer utilizes cervicography [10], a technique in which cervical images are taken at maximum magnification after applying 5% acetic acid to the cervix [11]. These images are then evaluated by human experts to identify any morphological abnormalities of the cervix. Nevertheless, this approach has constraints as it requires substantial people and material resources. For precise analysis of cervical dilatation tests, it is necessary to have a qualified professional reader and specialist photography equipment that can magnify the images by more than 50 times [12]. Moreover, the objectivity of the reader is limited and can only be improved by implementing systematic and frequent quality checks for readers. Now, there are potential errors made by both different observers and the same observer, but because there are no regular controls in place, there are limited data available on this issue. Moreover, the outcomes can differ depending on the subjective viewpoints of readers and the reading conditions [13,14].

In order to overcome these restrictions, computer-aided diagnostic methods, such as traditional machine learning (ML) and deep learning (DL), have been utilized to detect patterns that are significant for medical diagnosis [15,16]. ML functions as an advanced framework for DL, encompassing procedures that examine and collect knowledge from data prior to generating informed selections. Feature engineering in machine learning involves the elimination of redundant variables, a process that relies on the expertise of professionals to pre-select essential variables. On the other hand, deep learning addresses this limitation by enabling the system to acquire crucial characteristics without the need for pre-determined variables or human assumptions, thus improving its ability to adapt.

This study classifies numerous histological types of cervical cancer, such as dyskeratotic, koilocytotic, metaplastic, parabasal, and superficial–intermediate, by using image datasets and advanced deep learning models. This approach signifies a notable advancement in improving the accuracy and effectiveness of cervical cancer diagnosis. By utilizing these advanced technologies, the categorization of different forms of cervical cancer becomes more precise and shows potential for enhancing diagnostic capacities in clinical environments.

- Proposed a novel deep learning framework combining NFE and AutoInt models for automatic cervical cancer image classification.
- Utilized the pre-trained VGG16 model for high-quality feature extraction, enhancing feature extraction efficiency.
- Conducted an extensive evaluation of multiple machine learning classifiers (KNN, LGBM, ECT, etc.), achieving near-perfect classification accuracy.
- Optimized time efficiency, with models like LDA and ECT performing well in time-sensitive tasks.
- Employed a robust, well-labeled dataset and K-fold cross-validation to ensure model reliability and generalizability.

## 2. Literature Review

The landscape of cervical cancer diagnosis and classification has undergone a transformative shift with the advent of deep learning-based systems. The use of sophisticated computational techniques, notably deep learning models, has accelerated breakthroughs in the precise and efficient categorization of cervical cancer from medical imaging data in recent years. This review of the literature digs into the evolving domain of deep learning applications for cervical cancer classification, investigating the methodology, models, and outcomes that influence the current state of research in this vital field of healthcare. This study [17] systematically compares the efficacy of machine learning (ML) and deep learning (DL) models in identifying cervical cancer indications within cervicography images. A total of 4119 cervicography images were assessed for cervical cancer positivity or negativity using ResNet-50 for DL and ML models, namely XGB, SVM, and RF. The images featured squares with eliminated vaginal wall regions. Machine learning algorithms distilled ten crucial features from a comprehensive set of 300. The ROC analysis produced the following AUCs: ResNet-50 0.97 (CI 95% 0.949–0.976), XGB 0.82 (CI 95% 0.797–0.851), SVM 0.84 (CI 95% 0.801–0.854), and RF 0.79 (CI 95% 0.804–0.856). ResNet-50 showcased superior performance compared to the trio of machine learning models, achieving an AUC of 0.97—a noteworthy 0.15-point improvement (*p* < 0.05) over their collective average of 0.82. This work [18] describes a fully automated system for detecting cervixes and classifying cervical cancer in cervigram images. For automatic cervix detection and cervical tumor categorization, the pipeline employs two pre-trained deep learning models. The first model detects cervix regions quickly—1000 times faster than existing data-driven models—while maintaining an intersection of union (IoU) accuracy of 0.68. The second model employs lightweight convolutional neural network (CNN) models to classify cervical tumors using self-extracted characteristics. With an area under the curve (AUC) score of 0.82.

This paper [19] introduces a groundbreaking system for classifying pap smear images into seven distinct categories, aiming to aid in the automated diagnosis of cervical cell abnormalities. Leveraging ResNet101 for feature extraction, the system employs a Support Vector Machine (SVM) classifier to discern the seven image classes. Notably, the proposed system achieves 100% accuracy and sensitivity in distinguishing between normal cases, and it attains 100% accuracy in distinguishing between normal and abnormal cases. Furthermore, the system effectively classifies high-level abnormality cases with high accuracy, and it separately studies and classifies low-level abnormality into two classes, mild and moderate dysplasia, achieving approximately 92% accuracy. The system is constructed in a cascading manner with five polynomial SVM classifiers. During training, it achieves an overall accuracy of 100%, while in testing, the overall accuracy for all seven classes is approximately 92%, reaching an impressive overall accuracy of 97.3%. This research [20] investigates DL classification methods utilizing the SIPaKMeD pap smear image dataset, with the goal of establishing a standard for future classification algorithms. With this strategy, the ResNet-152 architecture obtained the highest classification accuracy, reaching 94.89%.

This research [21] aims to develop an automated system using Transfer Learning (TL) to identify cervical cancer subtypes cervical adenocarcinoma (CADC) and cervical squamous cell carcinoma (CSCC) from histopathology images. A dataset comprising 59 high-resolution Whole Slide Images (WSIs) was utilized, with 45 WSIs allocated for model training and 14 for testing. The proposed model achieved an accuracy of 85% and an AUC score of 86%. This work [22] presents DeepCervix, a DL-based hybrid deep feature fusion (HDFF) method for accurate cervical cell categorization. The suggested approach makes use of a variety of DL models to gather a wide range of data and improve classification performance overall. With respect to base DL models and the LF technique, HDFF performs better when tested on the SIPAKMED dataset. Notably, the two-class, three-class, and five-class classifications achieve classification accuracy rates of 99.85%, 99.38%, and 99.14%, respectively. Additionally, the approach obtains 98.32% accuracy for binary class and 90.32% accuracy for seven-class classification when tested on the Herlev dataset. Using the SIPaKMeD image dataset, [23] aims to create a classification model for cervical cell images by applying the CNN technique. In order to improve model accuracy, studies with the AlexNet architecture that uses a non-padding technique also add padding by inputting zero pixels. Empirical findings demonstrate that the addition of the padding technique to the AlexNet architecture improves model accuracy significantly, rising from 84.88% to 87.32%.

This work [24] introduces a hybrid deep model for detecting cervical cancer by classifying pap smear images. The Gaussian technique improves the original dataset. Within a hybrid architecture that includes Darknet53 and Mobilenetv2 models as the foundation, feature maps are retrieved from both the original and upgraded datasets. For dimension reduction, the feature maps are integrated and refined using Neighborhood Component Analysis (NCA). The optimized feature map is subsequently classified using several classifiers. The suggested hybrid model exceeds previous investigations, obtaining an accuracy rate of 98.90% with the Support Vector Machine (SVM) classifier. This paper [25] provides a CNN-based classification algorithm for cervical cells based on the SIPaKMeD dataset with five cell types. CNN distinguishes between healthy cells, precancerous cells, and benign cells. Pap smear images are segmented, and augmented cervical cell images are processed using a simplified CNN with four convolutional layers. The proposed CNN has a 91.13% accuracy, making it a simple yet effective method for cervical cell classification.

Despite the notable progress highlighted in the literature, it is important to acknowledge certain limitations within the existing studies. One recurring challenge is the availability and standardization of datasets. Many studies rely on specific datasets, such as SIPaKMeD or Herlev, which cannot fully capture the diversity and variability present in real-world clinical scenarios. Limited access to large, diverse datasets can impact the generalizability of the proposed models. A notable limitation in several studies is the relatively small sample sizes, which can impact the statistical robustness of the findings. Limited access to extensive datasets can constrain the ability to fully explore the nuances of cervical cancer variations and subtypes. Another common limitation lies in the interpretability of deep learning models. While these models often demonstrate impressive accuracy, understanding the underlying decision-making processes remains a challenge. See Table 1.

## 3. Materials and Methods

This study employed a methodology that was executed on Google Colab 0.0.1a2 using a T4 GPU with 16 GB of RAM [26]. The suggested approach was implemented using Colab Notebook 5.5.6 [26], a web-based interactive platform that seamlessly integrates live code execution, visualization, and explanatory text. The programming language chosen for this implementation was Python 3.10, and other libraries, including sci-kit-learn 1.5.2 and pandas 2.2.2, were utilized throughout the process. TensorFlow is a widely used open-source deep learning framework designed for the Python programming language. The platform provides a variety of tools for different applications such as classification, regression, and clustering [27].

### 3.1. Proposed Methodology

**Figure 1:**
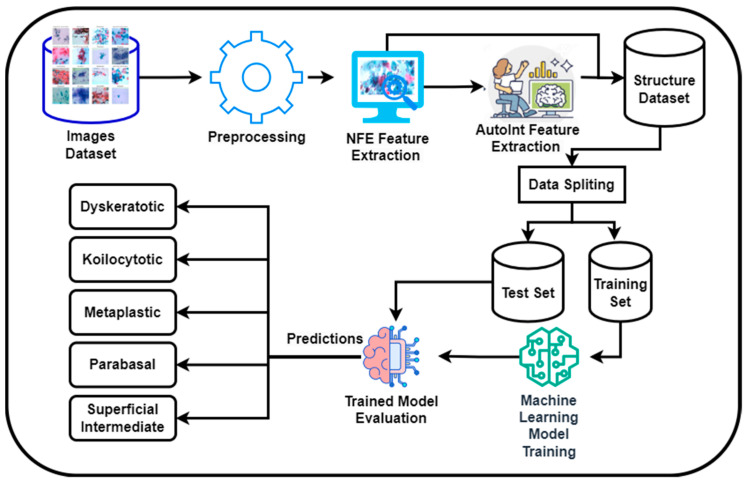
Proposed methodology diagram.

Figure 1 presents a novel methodology for the classification of cervical cancer images utilizing a deep learning approach on a publicly available dataset [28]. This framework comprises two primary models: the Neural Feature Extractor (NFE) and the Automatic Feature Interaction Learning via Self-Attentive Neural Networks (AutoInt) Model. Initially, the NFE model, built upon the VGG16 architecture with weights pre-trained on ImageNet and modified to exclude the top layer, processes the images to extract high-dimensional features. These features are then passed through a global average pooling layer to reduce dimensionality.

Subsequently, the extracted features are fed into the AutoInt model, a custom deep learning architecture designed to interpret complex interactions within the data, facilitating a more nuanced understanding of the underlying patterns significant for classification tasks. Both models operate in a sequential workflow where the output of the NFE serves as the input to the AutoInt model. After that, these features were then fed into machine learning models for classification.

### 3.2. Dataset

The dataset utilized in this study, retrieved from [28], comprises a substantial collection of 25,000 images. These images were meticulously captured using a Charge-Coupled Device (CCD) camera, meticulously modified to seamlessly integrate with an optical microscope. This tailored approach ensures the acquisition of precise depictions of squamous cells, contributing to the dataset’s robustness and relevance to cervical cancer analysis. Categorized into five distinct groups—dyskeratotic, koilocytotic, parabasal, metaplastic, and superficial-intermediate—the dataset captures a comprehensive spectrum of morphological characteristics inherent to cervical cells. Dyskeratotic cells, for instance, exhibit early and aberrant keratinization, distinguishing them by specific visual features. Koilocytotic cells, on the other hand, are characterized by vesicular nuclei, often observed in binucleated or multinucleated arrangements.

**Figure 2:**
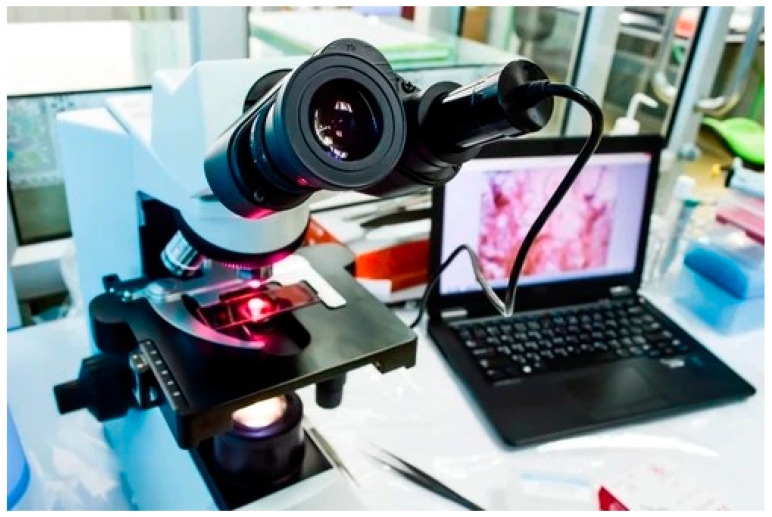
Microscope integrated with a CCD camera system for real-time data collection and analysis.

**Figure 3:**
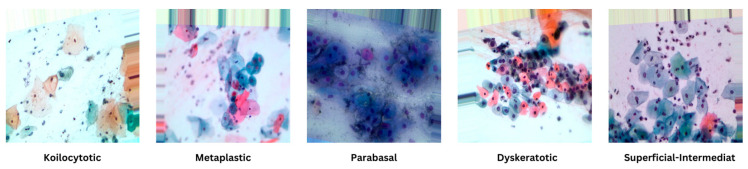
Sample images from the CCD camera.

**Figure 4:**
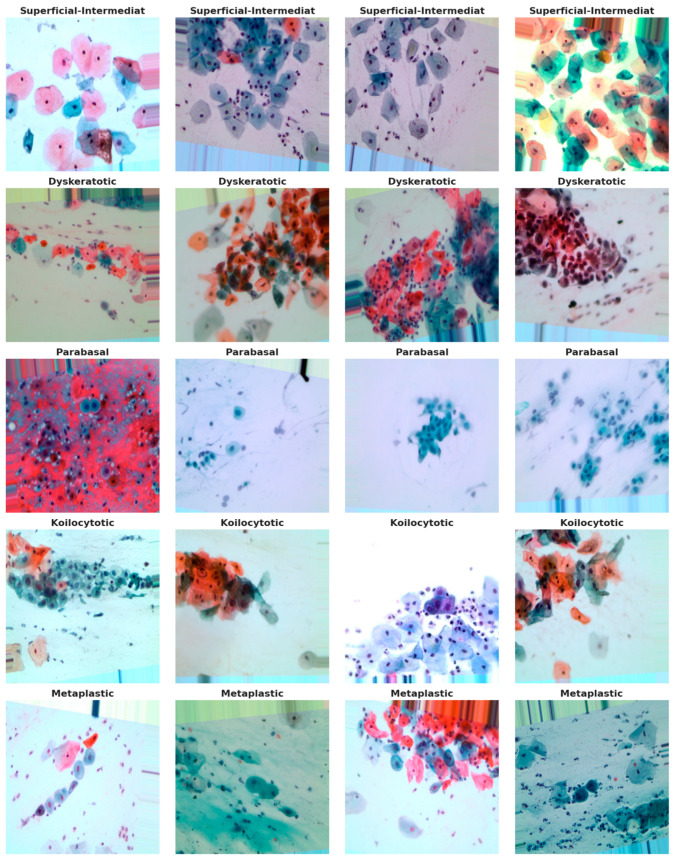
Sample images from the dataset.

To further enhance the dataset, we have collected an additional 1000 images per class from Sheikh Zayed Medical College, as shown in Figure 2, along with sample images from our own data collection in Figure 3 and from a Kaggle dataset in Figure 4. This augmentation strengthens the dataset’s diversity and improves its capacity for accurate classification and analysis.

**Figure 5:**
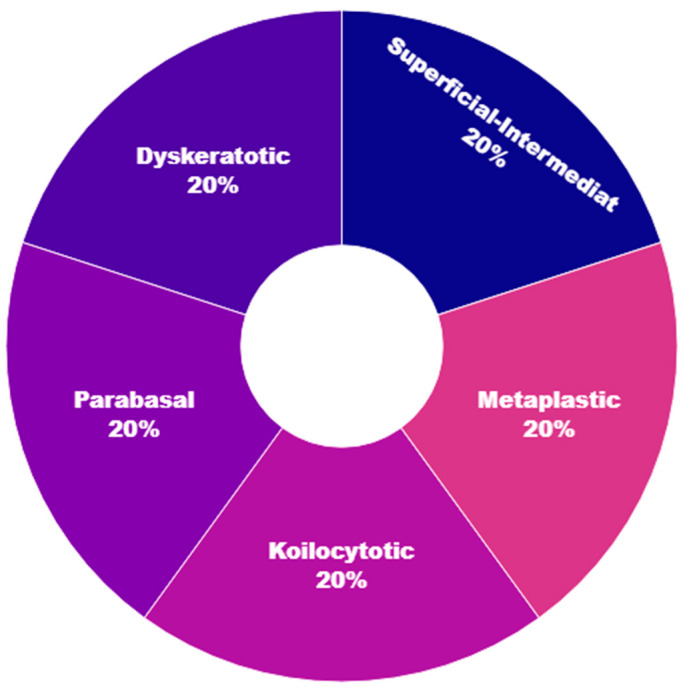
Distribution of labels in an image dataset.

Figure 5 serves as a visual representation, providing an illustrative glimpse into the diversity of sample images sourced from the dataset. This diverse and well-defined dataset, characterized by distinct morphological categories, forms the foundation for robust model training and evaluation in the subsequent stages of the research.

### 3.3. NFE Feature Extraction

The Neural Feature Extractor Model (NFEModel) is a customized neural network that leverages the architecture of the famous VGG16 model, with its focus on extracting features from images. This model modifies the VGG16 convolutional network, which is well-known for its high performance in classifying images, to focus on tasks that include analyzing and identifying complex patterns in images, such as medical imaging for cervical cancer. The NFEModel gains an advantage by utilizing the pre-trained weights obtained from the ImageNet database, which provides it with a strong and comprehensive knowledge of various visual content. It excludes the uppermost layer of the original VGG16 model to provide customization based on unique project requirements [29]. After applying these changed layers to the images, the model utilizes a Global Average Pooling (GAP) layer. This layer reduces the large amount of data from the previous convolutional layers by averaging down the spatial information, making it more manageable [30]. The simplified collection of features is now prepared for additional analysis or classification in following phases of the model, making the NFEModel an essential initial step in intricate image-based diagnosis and classification systems.

The model architecture described is designed for efficient feature extraction from images, using a pre-trained VGG16 model as its backbone. The VGG16 model, originally trained on the ImageNet dataset, has been shown to be highly effective for recognizing and analyzing common visual patterns in large-scale image datasets. In this model, the top layers responsible for classification are excluded (include_top = False), ensuring that the focus remains on the lower convolutional layers for feature extraction. The input to the model consists of images resized to 512 × 512 × 3, representing the height, width, and three-color channels (RGB).

The feature extraction process proceeds with a Global Average Pooling (GAP) layer, which significantly reduces the dimensionality of the feature maps produced by VGG16. Mathematically, for a 3D tensor x∈R^h×w×d^ representing the feature maps, where *h* and *w* are the spatial dimensions and *d* is the depth, the GAP layer computes the average of all values in each feature map. This can be expressed as follows:

(1) $$yk=\frac{1}{h\times x}\;\sum_{i=0}^{h}\sum_{j=1}^{w}x_{ij}k$$

This operation compresses spatial information, creating a lower-dimensional but representative feature vector that retains the most essential information from the input image [31].

Following the GAP layer, a dense (fully connected) layer with 256 neurons is applied. The dense layer performs the following transformation on the input *y*:

(2) $$z=f\left(w_{y}+b\right)$$

where w∈R^256×d^ is the weight matrix; b∈R^256^ is the bias vector; and *f* is the ReLU (Rectified Linear Unit) activation function. The ReLU function, defined as f(x) = max(0,x), introduces non-linearity, allowing the network to learn complex relationships between the input features [32].

To mitigate overfitting, the model includes a dropout layer with a dropout rate of *p* = 0.5. During training, dropout randomly sets a fraction *p* of the neurons to zero, as defined by:

(3) $${\hat{z}}_{i}=\left\{\begin{array}{c} z_{i}\;\mathrm{with}\;\mathrm{probability}\;(1-p)\;\\ 0\;\mathrm{with}\;\mathrm{probability}\;p \end{array}\right.$$

This technique helps the model generalize better by preventing it from relying too heavily on specific neurons or patterns in the data [33].

The final layer is another dense layer with 128 neurons, which further refines the extracted features. The mathematical operation is similar to the earlier dense layer, with the final output represented as:

(4) $$\hat{y}=f\left({\overset{´}{w}}_{\hat{z}}+\overset{´}{b}\right)\;\;$$

where w′∈R^128×256^ is the weight matrix, and b′∈R^128^ is the bias term.

**Figure 6:**
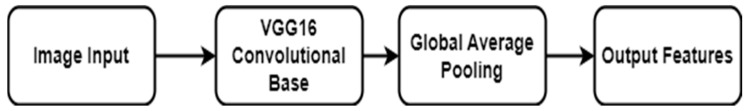
Architecture of the NFE model.

The data processing pipeline includes an Image Data Generator, which loads and preprocesses the images in batches. The images are resized to 512 × 512, and the preprocess input function normalizes pixel values for compatibility with VGG16, ensuring the images are in a format the model can process efficiently [29]. See Figure 6.

The use of powers of 2 for layer sizes is justified by its computational efficiency, particularly in GPU-based frameworks like TensorFlow 2.17.0 and PyTorch 2.5.0+cu121. These sizes align better with binary systems, leading to marginally improved memory usage and performance. Additionally, the powers of 2 provide a convenient scaling factor for hyperparameter tuning, simplifying experimentation and reducing the search space for model optimization. While not strictly necessary, this approach strikes a balance between performance and practical ease of use.

#### AutoInt Features Extraction

The AutoInt model is a neural network structure specifically created to understand and represent the complex connections between features in datasets with a large number of dimensions. AutoInt’s strength lies in its ability to automatically learn feature interactions through self-attention mechanisms, which are well-suited for capturing complex relationships between input features, especially in high-dimensional datasets like medical imaging. Unlike traditional models that rely on manual feature engineering, AutoInt identifies important feature interactions at multiple levels, focusing on the most relevant combinations. This leads to improved generalization and prediction performance. We will expand on this theoretical foundation to better explain how AutoInt enhances performance, particularly in tasks involving intricate patterns such as medical image classification. AutoInt utilizes attention processes to automatically identify and represent interactions at multiple levels instead of relying on manually designed feature interactions as traditional systems do. The fundamental concept underlying AutoInt is to employ self-attention layers, similar to those present in Transformer models, to acquire knowledge about the significance of interactions between pairs of features without relying on explicit manual feature engineering [34]. This strategy allows the model to concentrate on the most pertinent feature combinations, which could enhance prediction performance in tasks such as classification, regression, and recommendation systems.

AutoIntModel is designed for automatic feature interaction learning through a series of dense (fully connected) layers. It processes input data in a hierarchical fashion, gradually reducing the dimensionality while applying non-linear transformations, thereby enabling the model to capture complex relationships between input features. The use of dense layers in this model is fundamental for transforming and combining features, which is a critical step in many machine learning tasks, such as classification and regression.

The first layer of the model, referred to as dense layer 1, consists of 128 units (neurons) and utilizes the Rectified Linear Unit (ReLU) activation function. The dense layer can be mathematically described as follows:

(5) $$z_{1}=f\left(W_{1\cdot x}+b_{1}\right)\;$$

where W_1_∈R^128×d^ is the weight matrix; b_1_∈R^128^ is the bias vector; x∈R^d^ is the input vector of dimensionality *d*; and *f* represents the ReLU activation function, defined as f(z) = max (0, z). This operation applies a linear transformation to the input data, followed by the ReLU activation, which introduces non-linearity into the model. The non-linearity enables the model to learn complex feature interactions by allowing certain neurons to become inactive (outputting zero), depending on the value of their inputs [32].

Following dense layer 1, the model passes the output to dense layer 2, which consists of 64 units and similarly applies the ReLU activation function. This layer can be described mathematically as follows:

(6) $$z_{2}=f\left(W_{2\cdot z_{1}}+b_{2}\right)\;$$

where W_2_∈R^64×128^ and b_2_∈R^64^ are the weight matrix and bias vector, respectively. Dense layer 2 further refines the feature interactions learned in the first layer by applying another set of transformations and non-linear activations. This hierarchical structure, where each dense layer learns progressively more abstract representations of the input, is a standard approach in deep learning architectures for modeling complex patterns in data [35].

The final layer of the model, called the output layer, consists of 32 units and also utilizes the ReLU activation function. The transformation applied in the output layer is given by:

(7) $$z_{3}=f\left(W_{3\cdot z_{2}}+b_{3}\right)\;\;$$

where W_3_∈R^32×64^ and *b*_3_∈R^32^. The output of this layer is a 32-dimensional vector, representing the final feature interactions learned by the model. These interactions are the result of the sequential transformations and activations applied through the dense layers. Depending on the specific task, this output could be used for further processing, such as classification or regression, in subsequent layers or models.

**Figure 7:**
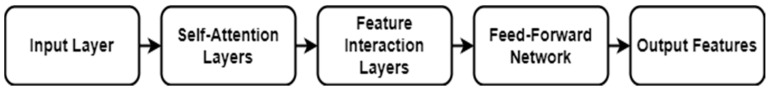
Architecture of the AutoInt model.

The use of dense layers for automatic feature interaction learning has been widely studied and found to be effective in capturing intricate patterns in data [36]. The ReLU activation function is particularly advantageous in such models due to its computational simplicity and ability to prevent gradient vanishing during backpropagation, a common problem in deep neural networks. The dense structure, combined with ReLU activations, facilitates efficient and scalable learning from high-dimensional input data. See Figure 7.

## 4. Results

This section presents the outcomes of the experiments and analyses conducted in this study. The findings are organized into two primary subsections: results from the NFE and AutoInt models, followed by the combined results of both approaches.

### 4.1. NFE Model Features Results

The dataset was divided into training and testing sets with a ratio of 70:30 to ensure that any model developed for cervical cancer image classification was robust. In this research, features for cervical image classification were extracted using a Neural Feature Extractor (NFE), and several classifiers were employed to classify these features, as shown in Table 2. Among the proposed models, the K-Nearest Neighbors (KNN) classifier was the best model, achieving the highest accuracy of 99.76% and demonstrating nearly perfect precision, recall, and F1-score. This indicates the efficiency of the proposed model in utilizing NFE features to enable accurate classification. The Light Gradient Boosting Model (LGBM) and Extra Trees Classifier (ECT) also yielded good results, with accuracies of 99.46% and 99.48%, respectively; both provided high reliability across all assessments. The Random Forest (RF) model achieved an accuracy of 99.18%, suggesting that ensemble methods can effectively handle a large number of features. With an accuracy of 92.56%, Logistic Regression (LR) also demonstrated good precision, recall, and F1-score, indicating solid performance with NFE features. The Calibrated Classifier (CV) and Linear Support Vector Classification (LSVC) were reliable as well, with accuracies of 90.84% and 91.84%, respectively, and they presented reasonable precision–recall curves. Despite a slight lag in performance, the Perceptron, Passive Aggressive Classifier (PAC), and Stochastic Gradient Descent (SGD) models showed commendable results, with accuracies ranging from 88.30% to 89.78%. On the other hand, the Ridge Classifier (RC), Ridge Classifier with Cross Validation (RCV), and Linear Discriminant Analysis (LDA) exhibited slightly lower performance, with accuracies between 87.32% and 88.18%. Finally, the Decision Tree Classifier (DTC) had the lowest accuracy of 87.42% among all models tested in this configuration.

### 4.2. AutoInt Model Features Results

In the cervical image classification task utilizing features extracted by a NFE and processed through an AutoInt model, various classifiers were evaluated to determine their effectiveness. From Table 3, it can be noted that the KNN classifier provided the best results in classifying cervical cancer images, achieving an overall accuracy of 94.44%, precision of 94.43%, recall of 92.06%, and F1-score of 93.09%. The ECT model followed closely with a prediction accuracy of 93.44%, and the RF model achieved a prediction accuracy of 92.04%. The LGBM demonstrated consistent performance with an accuracy of 91.66%, indicating that the model effectively learned the patterns when working with the extracted features. The proposed LR model produced a slightly lower accuracy of 72.72%, yet it maintained stability across other metrics such as precision, recall, and F1 score. The alternative models, including the CV and LSVC, yielded accuracy of 68.98% and 71.26%, respectively, which are considered reasonable. Other models, such as the Perceptron, PAC, and SGD, performed less favorably, with accuracy ranging from 56.82% to 70.12%. The Ridge RC and RCV produced accuracies of 68.70% and 68.74%, respectively. Consequently, the LDA model achieved an accuracy of 70.44%, which is reasonable considering that linear models often struggle with large datasets. Despite achieving an accuracy of 76.44%, the DTC ranked higher than some other models, although its stability of performance was not as strong as that of the previously discussed ensemble models.

### 4.3. Combined Features Results

The dataset was meticulously divided into training and testing sets in a 70:30 ratio, enabling a comprehensive evaluation of cervical cancer image classification models, as shown in Table 4. In this task, features extracted through an NFE, combined with AutoInt, were processed using various classifiers to assess their effectiveness. The KNN classifier achieved the highest performance with an accuracy of 99.69%, demonstrating exceptional ability to correctly classify images. Its precision, recall, and F1-score, all at 99.69%, indicate a strong balance between correctly identifying positive cases and minimizing both false positives and false negatives. The LGBM closely followed, delivering an accuracy of 99.36% and maintaining high precision, recall, and F1-score metrics, although marginally lower than the KNN. The ECT and RF also demonstrated robust performance, with accuracies of 99.40% and 98.91%, respectively, showing their ability to handle complex data patterns effectively. LR achieved an accuracy of 93.96% with excellent precision, recall, and F1 scores, demonstrating consistent and reliable classification. CV and Perceptron models performed well, achieving accuracies of 92.24% and 90.40%, respectively. The PAC, LSVC, and SGD models also exhibited solid results, with accuracies ranging from 89.67% to 93.26% while maintaining strong precision, recall, and F1-scores. The RC and its calibrated version RCV achieved slightly lower accuracies of 90.51% but still maintained consistent performance across all metrics. The LDA model provided reasonable results with an accuracy of 90.88%, despite the inherent challenges of linear models when working with complex datasets. The DTC had the lowest performance among the classifiers, with an accuracy of 88.01% and correspondingly lower scores in precision, recall, and F1-score.

**Figure 8:**
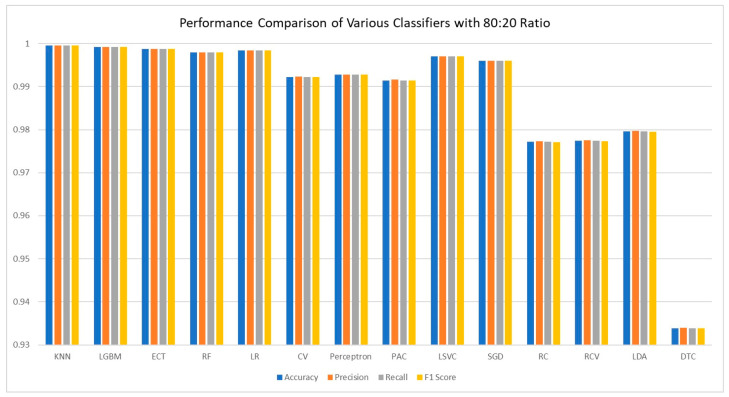
Visualization of comparison of various classifiers.

The dataset was meticulously divided into training, testing, and validation sets with an 80:20 ratio, allowing for a thorough evaluation of image classification models for cervical cancer. Both AutoInt and Neural Feature Extractor-related feature combinations were applied to compare the performance of different classifiers for cervical cancer image classification based on the results given in Table 5 and the corresponding visualization in Figure 8. Among the above models, the KNN came out as the best performing model, clearly depicting high accuracy, precision, recall, and an F1-score of 99.96%. This result proves the effectiveness of the KNN in managing the combined features by making sure that predictions made are very accurate with minimal errors. Next up, the LGBM provided equally impressive accuracy at 99.92% along with near-perfect scores for all other evaluation measures, proving that the model can excel in handling intricate data patterns effectively. Likewise, the ECT remained highly accurate with a reliability of 99.88% as the importance of ensemble methods was highlighted once again. The RF also boasted high accuracy of 99.80% with good balance between precision, recall, and F1 scores to support the reliability of ensemble-based models. Nevertheless, it was slightly worse for LR, with the accuracy of 99.84% being obtained through its stability being preserved across all of the significant values that prove that linear models are also effective with high-quality features only. The CV was the second with 99.22% accuracy, and it proved to have good generalization with the precision and recall measure, almost equal to its accuracy. Perceptron, PAC, and LSVC performed fairly well with accuracies of 99.28%, 99.14%, as well as 99.70%, respectively, which means they are capable of performing well though not at the same velocity as the recommended ensemble models. SGD also did quite well with 99.60% accuracy, thereby substantiating the fact that gradient-based optimization methods do learn well from the combined features. Here, it can be observed that the performance of the test was 97.72% along with RC, and the performance of RCV at the end of the fifth fold was also 97.74%, which indicates that the cross-validation did not affect the performance in a significant way. Despite the issue of LDA when dealing with non-linear data, it was able to obtain close to 97.96% accuracy, which is acceptable. However, the DTC obtained the lowest accuracy and cross-validation score of 93.38%, making it more susceptible to overfitting other methods like RF and ECT.

**Figure 9:**
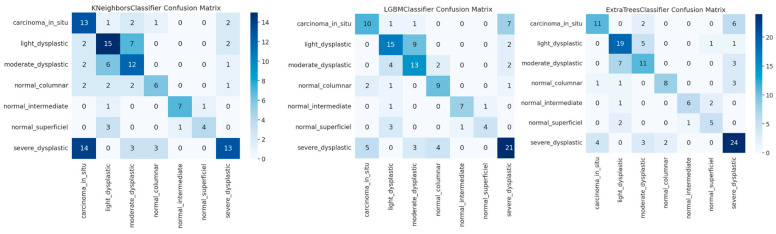
Visualization of ROC curve of best classifiers.

The confusion matrices in Figure 9 for KNN, LGBM, and ECT representing classification of cervical cancer images across multiple categories show the effectiveness of these models. KNN clearly shows excellent performance with very few misclassified, especially on the classification of severe dysplastic cases. However, there is some overlap, i.e., blocking hyperplastic cases from being misclassified as carcinoma suggests difficulties in discriminating very similar classes. High precision is maintained by the LGBM classifier, with confusion on moderate and low dysplastic cases showing the complexity of differentiating patterns similar to one another. From the ECT model, we further show that ensemble methods are powerful, as they can provide reasonable predictions even with a handful of misclassifications like severe or light dysplastic being occasionally predicted as carcinoma. The results are in agreement with the reported high accuracy, precision, recall, and F1 scores of all models, which included high accuracy scores of 99.96% for KNN and 99.92% for LGBM, followed by ECT at 99.88%. The confusion matrices demonstrate that all three models can well resolve imbalanced classes; however, ensemble-based methods such as ECT have superior generalization. The findings here underline that in order to maximize model performance, these advanced feature combinations, such as AutoInt and Neural Feature Extractors, need to be leveraged. This further validates the robustness for a complex medical image classification task of the models in being able to maintain their consistency in accuracy and low error rates on a variety of folds.

**Figure 10:**
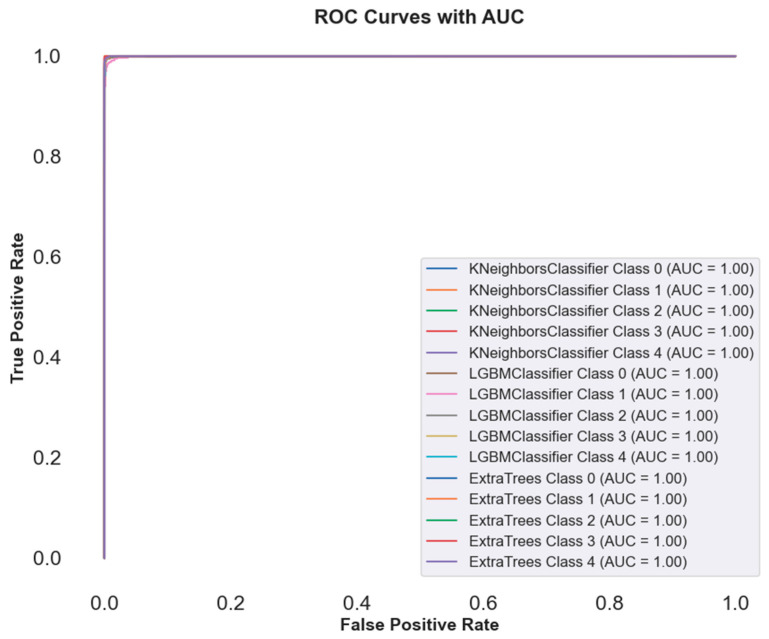
Visualization of ROC curve of best classifiers.

In Figure 10, an ROC curve plot compares the performance of some methods on cervical cancer image classification using AutoInt and Neural Feature Extractor features as a combination. Near-perfect AUC values (1.00) of the ROC curve for the best three classifiers for multiple classes indicate the best classification performance. On all classes, ECT, LGBM, and KNN classifiers show perfect discrimination, indicating the robustness of these models to complex feature sets. Curves overlapped, which means that these classifiers must have almost similar performance in discriminating between positive and negative instances. Table 5 reports that the high AUC values indicate that ensemble methods and feature extraction techniques effectively increased model accuracy, precision, recall, and F1 scores.

**Figure 11:**
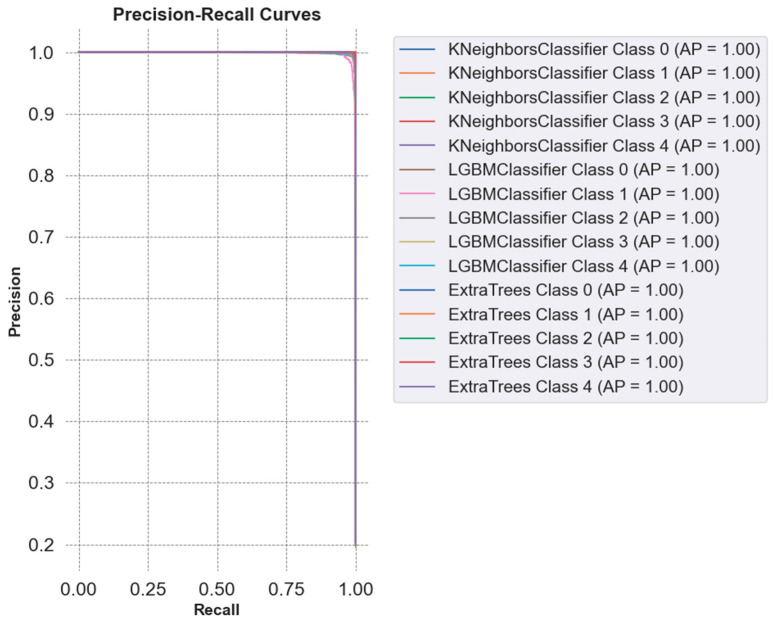
Visualization of precision–recall curves of best classifiers.

Figure 11 presents the precision–recall curves for the comparison of classifiers in cervical cancer image classification using combined feature sets (AutoInt + Neural Feature Extractor). The best three classifiers, KNN, LGBM, and ETC, achieved an average precision (AP) of 1.00 across all classes, signifying near-perfect precision and recall values. This result indicates that the models consistently identify true positives without generating false positives or false negatives, further highlighting their efficacy in managing the intricate patterns of the dataset.

## 5. K-Fold Validations

K-fold cross validation was carried out to assess the generalizability of ML models. Cross-validation is a widely used technique in ML and statistical analysis to assess how well a model can generalize and perform overall. The cross-validation technique involves repetitively training and assessing a model using different combinations of folds as the training and testing datasets. The cross-validation results are displayed in Table 6.

The comparative assessment of several classifiers using five-fold cross-validation emphasizes their unique performance in terms of accuracy and standard deviation. The KNN and LGBM models demonstrated outstanding performance, with validation accuracies of 99.975% and 99.935%, respectively. These results were accompanied by minimal standard deviations, demonstrating consistent and dependable classification capabilities. The ECT and RF classifiers demonstrated exceptional accuracies of 99.88% and 99.79%, respectively. These accuracies remained consistent over multiple folds, highlighting the stability of these classifiers in accurately classifying cervical cancer images. The LSVC and SGD models exhibited robust performance, achieving accuracy just around 99.5%. On the other hand, more simpler models like LR, CV, and Perceptron all produced satisfactory results, although with slightly greater variability, especially for the Perceptron. At the lower end, RC, RCV, and LDA demonstrated satisfactory accuracy of approximately 98%. However, their greater standard deviations suggest discrepancies in their performance across different datasets. The Decision Tree Classifier (DTC) had a notable delay, achieving an accuracy of 92.165% and displaying the greatest amount of variation. This indicates that it may be less appropriate for applications that demand high levels of precision and stability. This investigation highlights the superiority of complex ensemble models such as LGBM and RF in achieving high and consistent classification accuracies. It also demonstrates the trade-offs between model complexity and performance reliability.

**Figure 12:**
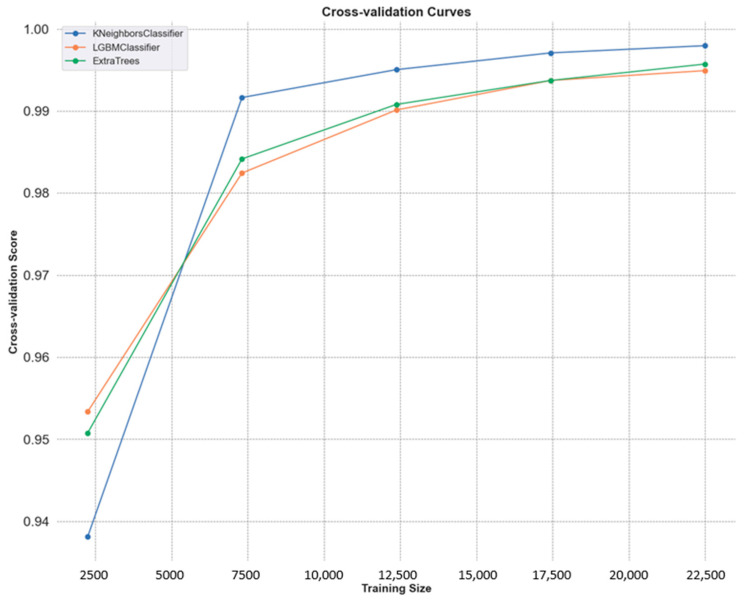
Cross-validation curves of KNN, LGBM, and ECT with increasing training size.

Figure 12 presents the cross-validation curves for the top-performing models: KNN, LGBM, and ECT. All three models are slightly worse as the training size drops, with KNN showing the highest performance and approaching perfect scores for all of them. The LGBM and ECT produce strong, stable accuracy as data increase, the robustness of which is demonstrated. We observe that curves converge at larger datasets, which means that all models are good generalizers; KNN remains slightly better than others, thus validating its power in cervical cancer image classification.

## 6. Prediction Time

Table 7 presents an intricate examination of the time effectiveness of several machine learning models in forecasting a single image after undergoing a feature extraction procedure. This assessment enables a straightforward comparison of the computational speed of various models, emphasizing how the simplicity or complexity of each model affects its prediction performance.

The results clearly demonstrate that simpler models often achieved faster prediction times. More precisely, the LDA algorithm demonstrated exceptional speed, completing predictions in just 3.33 s. This highlights its efficiency in situations where rapid decision-making is of utmost importance. The ECT and LSVC exhibited exceptional efficiency, with prediction times of 4.47 and 4.5 s, respectively, closely trailing by the LGBM at 4.56 s. In contrast, many intricate or computationally demanding models like CV and SGD had longer prediction times, clocking in at 9.36 and 9.72 s, respectively. The prediction times for models such as RC and RCV were also slower, with durations of 8.72 and 8.33 s, respectively. The DTC, commonly seen as a relatively uncomplicated model, demonstrated a moderate prediction time of 7.87 s, which may still be achievable for many applications but is slower compared to some of its less complex alternatives. These findings highlight the significance of taking into account both precision and computing speed when choosing a model for practical use, particularly in time-critical settings. The careful consideration and management of these parameters can have a substantial effect on the effective implementation of machine learning models in different situations.

### 6.1. Comparison with Existing Studies

The current study stands out from previous works by achieving remarkable accuracy across various machine learning models while applying them to different cervical cancer screening datasets, as shown in Table 8. This research demonstrates that traditional machine learning models such as KNN, LGBM, Extra Trees, Random Forest, and Logistic Regression can outperform or match the performance of deep learning models used in prior studies. For example, when applied to the custom cervical cancer dataset in this study, KNN achieved an impressive accuracy of 99.96%, followed closely by LGBM (99.92%) and Extra Trees (99.88%). These results are significantly higher than those reported in earlier studies, where complex models like ResNet and CNN were utilized. For instance, in study [17], ResNet-50 achieved 97% accuracy, while models like XGB, SVM, and RF achieved between 79% and 84%. Similarly, a study [24] reported 98.9% accuracy using a hybrid deep learning model on pap smear images. In contrast, the current study achieves superior performance using simpler models with lower computational complexity.

A key difference between the current study and previous works lies in the diversity of the datasets and the types of images involved. Previous studies used datasets such as cervicography images, pap smear images, and histopathology whole-slide images (WSIs), each varying significantly in image quality, resolution, and cell types. For example, the SIPaKMeD dataset used in studies [20,25] contains cytological images from pap smears, focusing on classifying five different types of cervical cells. In contrast, the Herlev dataset used in study [22] contains cell images from different stages of cervical cancer. The variance in image types, from digital cervicography to cytological slides, impacts model performance differently, requiring careful tuning of techniques for each dataset.

In this context, the current study demonstrates the adaptability of its machine learning models by achieving competitive results across various image types. When tested on the SIPaKMeD dataset, models such as LGBM and Extra Trees achieved accuracies of 91.11% and 88.89%, respectively, while on the Herlev dataset, the same models performed with accuracies of 61.24% and 65.11%. Though these numbers are slightly lower than the performance on the custom dataset, they still remain close to the top results from prior studies (e.g., 99.14% for the SIPaKMeD dataset in study [22]). This demonstrates that the models are capable of handling different types of cervical images effectively.

### 6.2. Discussion

In this study, the KNN classifier outperformed other models, achieving an impressive accuracy of 99.96%, followed closely by the LGBM classifier with 99.92%. The high performance of KNN can be attributed to its simplicity and its effectiveness in handling the feature-rich data extracted by the Neural Feature Extractor (NFE). KNN’s ability to capture subtle variations in cervical cell morphology is crucial for accurate classification. Similarly, LGBM performed exceptionally well due to its gradient-boosting approach, which allows it to handle large datasets and complex patterns effectively. Ensemble methods like Extra Trees and Random Forest also showed strong performance, with accuracies of 99.88% and 99.80%, respectively, benefiting from their ability to reduce overfitting by averaging multiple decision trees. However, despite these strong results, it is important to discuss potential limitations, particularly concerning the dataset size and variability. The datasets used in this study may not fully reflect the diversity of real-world clinical settings, where image quality, resolution, and cell morphology can vary widely. A smaller or less diverse dataset may limit the generalizability of the model, as it can overfit the specific patterns seen in the training data, making it less robust when exposed to new or unseen data. Additionally, while the feature extraction process using a pre-trained VGG16 architecture and the AutoInt model contributed to the strong performance, the reliance on pre-trained weights from non-medical domains might limit the system’s adaptability to specialized medical imaging tasks. To address these limitations and improve generalizability, further work includes testing the model on larger, more diverse datasets and incorporating real-world variability in cervical cancer images. This would provide a clearer understanding of how the model performs in different clinical scenarios. Additionally, fine-tuning the feature extraction model on domain-specific datasets could improve the system’s ability to handle the unique challenges posed by medical images.

## 7. Conclusions

Cervical cancer poses a significant global health challenge, causing substantial mortality, with over half a million women diagnosed annually. As the second most prevalent cancer among women worldwide, it bears a 60% mortality rate, disproportionately affecting resource-limited regions. The research introduced a sophisticated method combining the Neural Feature Extractor (NFE) and the AutoInt model for deep learning-based image classification. The NFE model, leveraging the VGG16 architecture with modifications for feature extraction tailored to cervical cancer cells, paired with the AutoInt model’s capability to understand complex feature interactions, has demonstrated high efficacy in classifying diverse morphological types of cervical cells. The evaluation of various machine learning classifiers on this dataset underscores the effectiveness of this combined approach. Specifically, the K_Neighbors Classifier (KNN) and the Light Gradient Boosting Machine (LGBM) emerged as top performers, showing exceptionally high accuracies, indicating their potential for reliable, real-world applications. The robustness of these classifiers was further validated through extensive K-fold cross-validation, confirming their generalizability and consistent performance across multiple subsets of data. In computational terms, the assessment of predictive efficiency showed that simpler models such as the LDA, ECT, and LSVC not only provided swift predictions but also maintained high accuracy, emphasizing the importance of selecting models that balance speed and precision, especially in clinical settings where timely decision-making is crucial. Looking forward, this study highlights the potential for these advanced machine learning models to revolutionize cervical cancer diagnostics. Future research could focus on further refining these models to enhance their accuracy and reduce computational costs. Additionally, expanding the dataset and incorporating real-world patient data might help improve the models’ applicability and robustness, ultimately leading to better clinical outcomes for patients worldwide. This continuous exploration of innovative deep learning architectures and their integration into medical diagnostics could pave the way for more effective and accessible cervical cancer screening and diagnosis tools.

## Figures and Tables

**Table 1:** Comparison of machine learning and deep learning models for cervical cell image classification—accuracy rates across various studies.

Study	Model Used	Accuracy (%)	Dataset
Study [17]	ResNet-50, XGB, SVM, RF	97 (ResNet-50), 82 (XGB), 84 (SVM), 79 (RF)	Cervicography images (4119)
Study [18]	Pre-trained DL models	82	Cervigram images
Study [19]	ResNet101, SVM	100 (SVM, normal vs. abnormal), 97.3 (overall)	Pap smear images
Study [20]	ResNet-152	94.89	SIPaKMeD pap smear images
Study [21]	Transfer Learning	85	Histopathology images (59 WSIs)
Study [22]	DL-based hybrid deep feature fusion	99.85 (two-class), 99.14 (five-class)	SIPAKMED and Herlev datasets
Study [23]	CNN (AlexNet with padding)	87.32 (AlexNet with padding)	SIPaKMeD image dataset
Study [24]	Hybrid model (Darknet53 + Mobilenetv2)	98.9 (SVM)	Pap smear images
Study [25]	CNN (four-layer simplified)	91.13	SIPaKMeD dataset (five cell types)
Current Study	KNN, LGBM, ECT, RF	99.96 (KNN), 99.92 (LGBM), 99.88 (ECT)	Public dataset (25,000 images)

**Table 2:** Evaluation of cervical cancer image classification models using NFE features.

Model	Accuracy	Precision	Recall	F1 Score
KNN	0.9876	0.987601	0.9876	0.9876
LGBM	0.9846	0.984599	0.9846	0.984598
ECT	0.9848	0.984834	0.9848	0.984804
RF	0.9818	0.981822	0.9818	0.981802
LR	0.9156	0.915554	0.9156	0.915518
CV	0.8984	0.900102	0.8984	0.896787
Perceptron	0.873	0.872319	0.873	0.872509
PAC	0.8584	0.858946	0.8584	0.858391
LSVC	0.9084	0.908125	0.9084	0.908179
SGD	0.8878	0.887576	0.8878	0.887574
RC	0.8636	0.863003	0.8636	0.862293
RCV	0.8632	0.862582	0.8632	0.861881
LDA	0.8718	0.872163	0.8718	0.871663
DTC	0.8642	0.864371	0.8642	0.864253

**Table 3:** Evaluation of cervical cancer image classification models using AutoInt.

Model	Accuracy	Precision	Recall	F1 Score
KNN	0.9344	0.934332	0.9344	0.934249
LGBM	0.9066	0.906647	0.9066	0.906348
ECT	0.9244	0.924658	0.9244	0.924304
RF	0.9104	0.910509	0.9104	0.910246
LR	0.7172	0.714116	0.7172	0.715063
CV	0.6798	0.672004	0.6798	0.673614
Perceptron	0.5868	0.595006	0.5868	0.587369
PAC	0.5582	0.562794	0.5582	0.553489
LSVC	0.7026	0.698711	0.7026	0.696961
SGD	0.6912	0.691049	0.6912	0.684251
RC	0.677	0.67573	0.677	0.668297
RCV	0.6774	0.676135	0.6774	0.668681
LDA	0.6944	0.692181	0.6944	0.69229
DTC	0.7544	0.762589	0.7544	0.762915

**Table 4:** Evaluation of cervical cancer image classification models using combined features with 70:30 ratio.

Model	Accuracy	Precision	Recall	F1 Score
KNN	0.9969	0.9969	0.9969	0.996933
LGBM	0.9936	0.993618	0.9936	0.993596
ECT	0.994	0.994024	0.994	0.993995
RF	0.989067	0.989101	0.989067	0.989049
LR	0.9396	0.939347	0.9396	0.939392
CV	0.9224	0.923667	0.9224	0.92081
Perceptron	0.904	0.903875	0.904	0.903669
PAC	0.896667	0.89787	0.896667	0.896828
LSVC	0.932667	0.932235	0.932667	0.932195
SGD	0.9168	0.916704	0.9168	0.916729
RC	0.905067	0.904232	0.905067	0.904121
RCV	0.905067	0.904215	0.905067	0.904109
LDA	0.9088	0.908571	0.9088	0.90842
DTC	0.880133	0.880111	0.880133	0.880086

**Table 5:** Evaluation of cervical cancer image classification models using combined features with 80:20 ratio.

Model	Accuracy	Precision	Recall	F1 Score
KNN	0.9996	0.9996	0.9996	0.9996
LGBM	0.9992	0.9992	0.9992	0.9992
ECT	0.9988	0.9988	0.9988	0.9988
RF	0.998	0.998	0.998	0.998
LR	0.9984	0.9984	0.9984	0.9984
CV	0.9922	0.9923	0.9922	0.9922
Perceptron	0.9928	0.9928	0.9928	0.9928
PAC	0.9914	0.9917	0.9914	0.9914
LSVC	0.997	0.997	0.997	0.997
SGD	0.996	0.996	0.996	0.996
RC	0.9772	0.9773	0.9772	0.9771
RCV	0.9774	0.9775	0.9774	0.9773
LDA	0.9796	0.9797	0.9796	0.9795
DTC	0.9338	0.934	0.9338	0.9339

**Table 6:** Cross-validation results.

Model	Validation Accuracy	Validation Accuracy STD
KNN	0.99975	0.000387
LGBM	0.99935	0.000339
ECT	0.9988	0.00062
RF	0.9979	0.000644
LR	0.99775	0.000447
CV	0.9922	0.001239
Perceptron	0.9933	0.002799
PAC	0.994	0.000689
LSVC	0.99495	0.00062
SGD	0.99425	0.001696
RC	0.98045	0.002384
RCV	0.9801	0.002206
LDA	0.98045	0.002199
DTC	0.92165	0.005733

**Table 7:** Predictive efficiency assessment: prediction time for machine learning algorithms.

Model	Time (s)
KNN	6.12
LGBM	4.56
ECT	4.47
RF	6.99
LR	5.44
CV	9.36
Perceptron	5.91
PAC	5.92
LSVC	4.5
SGD	9.72
RC	8.72
RCV	8.33
LDA	3.33
DTC	7.87

**Table 8:** Comparison with existing studies.

Study/Model	Dataset	Model(s)	Accuracy (%)
Study [17]	Cervicography images (4119)	ResNet-50, XGB, SVM, RF	97 (ResNet-50), 82 (XGB), 84 (SVM), 79 (RF)
Study [18]	Cervigram images	Pre-trained DL models	82
Study [19]	Pap smear images	ResNet101, SVM	100 (SVM, normal vs. abnormal), 97.3 (overall)
Study [20]	SIPaKMeD pap smear images	ResNet-152	94.89
Study [21]	Histopathology images (59 WSIs)	Transfer Learning	85
Study [22]	SIPAKMED and Herlev datasets	DL-based hybrid deep feature fusion	99.85 (two-class), 99.14 (five-class)
Study [23]	SIPaKMeD image dataset	CNN (AlexNet with padding)	87.32
Study [24]	Pap smear images	Hybrid model (Darknet53 + Mobilenetv2)	98.9 (SVM)
Study [25]	SIPaKMeD dataset (5 cell types)	CNN (four-layer simplified)	91.13
Current Study	Kaggle Cervicography dataset	KNN, LGBM, ETC, RF, LR	48.15 (KNN), 55.89 (LGBM), 51.51 (ETC), 52.86 (RF), 55.89 (LR)
Current Study	SIPaKMeD dataset	KNN, LGBM, ETC, RF, LR	85.80 (KNN), 91.11 (LGBM), 88.89 (ETC), 87.77 (RF), 86.04 (LR)
Current Study	Herlev dataset	KNN, LGBM, ETC, RF, LR	54.26 (KNN), 61.24 (LGBM), 65.11 (ETC), 64.34 (RF), 60.47 (LR)
Current Study	Cervical Cancer dataset	KNN, LGBM, ETC, RF, LR	99.96 (KNN), 99.92 (LGBM), 99.88 (ETC), 99.80 (RF), 99.84 (LR)

## Data Availability

The original contributions presented in the study are included in the article, further inquiries can be directed to the corresponding author.
